# Epigenetic Regulation of Werner Syndrome Gene in Age-Related Cataract

**DOI:** 10.1155/2015/579695

**Published:** 2015-10-05

**Authors:** Xi Zhu, Guowei Zhang, Lihua Kang, Huaijin Guan

**Affiliations:** Eye Institute, Affiliated Hospital of Nantong University, Nantong, Jiangsu 226001, China

## Abstract

*Purpose*. To examine the promoter methylation and histone modification of WRN (Werner syndrome gene), a DNA repair gene, and their relationship with the gene expression in age-related cataract (ARC) lens. *Methods*. We collected the lenses after cataract surgery from 117ARC patients and 39 age-matched non-ARC. WRN expression, DNA methylation and histone modification around the CpG island were assessed. The methylation status of Human-lens-epithelium cell (HLEB-3) was chemically altered to observe the relationship between methylation and expression of WRN. *Results*. The WRN expression was significantly decreased in the ARC anterior lens capsules comparing with the control. The CpG island of WRN promoter in the ARC anterior lens capsules displayed hypermethylation comparing with the controls. The WRN promoter was almost fully methylated in the cortex of ARC and control lens. Acetylated H3 was lower while methylated H3-K9 was higher in ARC anterior lens capsules than that of the controls. The expression of WRN in HLEB-3 increased after demethylation of the cells. *Conclusions*. A hypermethylation in WRN promoter and altered histone modification in anterior lens capsules might contribute to the ARC mechanism. The data suggest an association of altered DNA repair capability in lens with ARC pathogenesis.

## 1. Introduction

Age-related cataract (ARC) is one of the dominant causes of visual impairment in the elderly [1]. The disease can be classified as cortical, nuclear, and posterior subcapsular according to the location of the opacity within the lens [2]. ARC is a complex disease with multiple genetic and environmental risk components, including UV light, sun exposure, vitamin C deficiency, and hypertension [3], but its etiology is not fully understood [4, 5].

Oxidative stress has long been recognized as an important mediator of pathophysiology in lens epithelial cells (LECs) and also plays a vital role in the pathogenesis of cataract [6–8]. Recent studies have reported the association between reactive oxygen species- (ROS-) induced DNA damage of LECs and the development of cataract [8–10]. In our previous studies, we have also found that oxidative DNA damage marker, 8-oxoG, was significantly increased in ARC group compared with control group [11, 12]. Oxidative DNA lesions are repaired by nucleotide excision repair, double-strand break (DSB) repair, and base excision repair [13, 14]. The WRN gene plays an important role in aging [15] and is known to function in repair of damaged DNA, particularly in repairing double-strand breaks [16]. The WRN protein belongs to RecQ family and has 1432 amino acids possessing both 3′→5′ DNA helicase and 3′→5′ DNA exonuclease activities. These biochemical functions are known to have roles in DNA replication, repair of DNA damage, gene transcription, and telomere maintenance [17]. WRN disruption causes Werner's syndrome (WS), an autosomal recessive segmental progeroid syndrome that results in accelerated aging and affects multiple organs and tissues [17]. Most WS patients develop bilateral ocular cataract when they are 20 years old and beyond [18, 19]. Previously, we reported that polymorphisms and copy number variations of WRN are associated with ARC [20, 21].

Epigenetics pertains to heritable alterations in gene expression that do not involve modification of the underlying genomic DNA sequence [22]. DNA methylation and histone modifications (including methylation, acetylation, sumoylation, and phosphorylation) are the major epigenetic mechanisms for gene expression [23]. Hypermethylation of promoter CpG islands and histone H3 methylated at lysine 9 have been linked to heterochromatin and gene silencing, whereas histone H3 acetylated is enriched in euchromatic domains and correlates with active gene expressions [24–26]. DNA methylation is a critical regulator of gene expression in the eye and is necessary for the proper development and postmitotic survival of retinal neurons [27]. Aberrant methylation patterns have been associated with age-related macular degeneration, cataract, pterygium, and retinoblastoma [28–32]. Changes in histone modifications have also been observed in experimental models of diabetic retinopathy and glaucoma [22]. A decreased expression of WRN is related to aberrant DNA hypermethylation in various tumors; epigenetic inactivation of this gene may be a biomarker for selection of drugs for the treatment of cancer [33–37].

In this study, we investigated WRN expression in anterior lens capsules and lens cortex of ARC and age-matched controls and analyzed the correlation between epigenetic modification and expression profiles of WRN gene to explore the possible effect of epigenetics on the development of ARC.

## 2. Materials and Methods

### 2.1. Study Participants

The research followed the tenets of the Declaration of Helsinki. All participants signed the informed consent forms. The study was approved by the Ethics Committee of Affiliated Hospital of Nantong University.

We enrolled 117 ARC patients that consisted of three subgroups: age-related cortical cataract (ARC-C), age-related nuclear cataract (ARC-N), and age-related posterior subcapsular cataract (ARC-P). The criteria for ARC group included (1) opaque ocular lenses, (2) ≥50 years of age, and (3) *C* ≥ 4, *N* ≥ 4, and *P* ≥ 4 according to the lens opacity classification system III (LOCSIII) [38] and excluded (1) complicated cataract due to high myopia, uveitis, ocular trauma, or other known causes and (2) hypertension, diabetes, or other systemic diseases. We enrolled 39 patients with vitreoretinal diseases who received transparent lens extraction as control group. The criteria for the control included (1) transparent ocular lenses and (2) ≥50 years of age and excluded (1) other major eye diseases such as glaucoma, myopia, diabetic retinopathy, and uveitis and (2) hypertension, diabetes, or other systemic diseases. The demographic information for all participants was listed in Table 1.

### 2.2. Anterior Lens Capsules and Lens Cortex Preparation

Anterior lens capsules that included lens epithelium (LE) and peripheral cortex were obtained through small incision extra capsular cataract surgery. The tissue was dissected and rapidly frozen in liquid nitrogen and then stored at −80°C for protein, RNA, and genomic DNA extraction.

### 2.3. RNA Isolation and cDNA Preparation

Total RNA from anterior lens capsules and lens cortex was isolated from the frozen tissues using TRIzol reagent (Invitrogen, Carlsbad, CA), and cDNAs were synthesized using PrimeScript RT reagent Kit (TaKaRa, Dalian, China).

### 2.4. Quantification of WRN mRNA

TaqMan gene expression assay probe (Applied Biosystems, Foster City, CA) was used for WRN mRNA quantification (Applied Biosystems assay ID: Hs01087915_m1). The results were normalized against the expression of housekeeping gene GAPDH from the same sample. RT-PCR was performed using ABI 7500 real time PCR system (Applied Biosystems, Foster City, CA). The fold change of WRN expression was determined using the comparative CT (2^−ΔΔCT^) method.

### 2.5. Western Blot Assay

The protein of anterior lens capsules and lens cortex was extracted in lysis buffer (1 M Tris-HCl at pH 7.5, 1% Triton X-100, 1% Nonidet p-40, 10% SDS, 0.5% sodium deoxycholate, 0.5 M EDTA, 10 mg/mL leupeptin, 10 mg/mL aprotinin, and 1 mM phenylmethylsulfonyl fluoride). Proteins were size-fractionated by sodium dodecyl sulfate-polyacrylamide gel electrophoresis and transferred onto polyvinylidene difluoride filter membranes (Millipore, Bedford, MA). Nonspecific protein binding to the membrane was blocked with blocking buffer (5% nonfat milk, 200 mM NaCl, 50 mM Tris, and 0.05% Tween 20). The blocked membrane was then incubated with mouse anti-human-WRN (1 : 800; Abcam, Cambridge, UK) and anti-GAPDH (1 : 1000; Santa Cruz, CA, USA) at 4°C for 18 h. The membrane was washed three times with TBST (20 mM Tris, 500 mM NaCl, and 0.1% Tween 20) for 5 min each time, followed by incubating with an alkaline phosphatase-conjugated goat anti-mouse IgG antibody (1 : 2000; Santa Cruz, CA, USA) for 2 h. Detection was performed using an ECL chemiluminescence kit (Pierce, Rockford, IL) and the signal was exposed to an X-ray film that was scanned using Image Quant software (Molecular Dynamics, Sunnyvale, CA, USA).

### 2.6. DNA Methylation Detection

DNA sequence of WRN from the NCBI genome database was used for the bioinformatic analysis. Transcription start site (TSS) of the gene was predicted by the online database (http://dbtss.hgc.jp/). CpG islands of WRN were predicted by using online software (http://www.urogene.org/methprimer/).

Genomic DNA from the frozen tissues and HLEB-3 was isolated by phenol/chloroform and ethanol extraction. Two micrograms of genomic DNA was treated with sodium bisulfite using the EpiTect Bisulfite Kit (Qiagen, Inc., Frederick, MD). EpiTect Control DNA (Qiagen, Inc.) was used as the positive controls in all experiments.

The bisulfite-sequencing PCR (BSP) primer was designed by web-based Meth Primer software (http://www.urogene.org/methprimer/) to cover a CpG island near WRN. The primers used for region 1 were 5′-TTATTTTGAAAGAAGTTTTTTTTGG-3′ (forward) and 5′-AAACAAACTATTATCCTCCCAACAC-3′ (reverse). The primers used for region 2 were 5′-TTTTTTGTGTTGGGAGGATAATAGT-3′ (forward) and 5′-AACAAAAAACAAAACTCCAAAAAAA-3′ (reverse). The primers used for region 3 were 5′-AGGTCTCCAGCCGGCGGGCACTCA-3′ (forward) and 5′-TGAGGGGAAGAGGGGGTC-3′ (reverse). The primers used for region 4 were 5′-TTTAGTGTATTTTTTGATTGAAGTT-3′ (forward) and 5′-CTAAACAACTAAAATCCTACATCCC-3′ (reverse). The PCR products were gel-extracted and cloned into the pMD-20-T vector (Takara, Japan). Plasmid-transformed bacteria DH5a were grown for 14 h and the plasmid DNA was isolated. At least 10 clones were chosen for sequence analysis. The degree of methylation was presented as mC/CpG.

### 2.7. Chromatin Immunoprecipitation (ChIP) Assay

Chromatin immunoprecipitation (ChIP) assay was performed using Tissue Acetyl-Histone H3 ChIP kit and Tri-Methyl-Histone H3K9 ChIP kit (Epigentek, Farmingdale, NY, USA) according to the manufacturer's instructions. Briefly, the anterior lens capsules and cortex were cross-linked with 1% formaldehyde for 8 min and then homogenized. The homogenate was sonicated for 4 pulses of 15 sec each at level 2 using the microtip probe of a Branson Digital Sonifier (Model 450, Branson Ultrasonics Corporation, Connecticut, USA), followed by a 40 sec interval on ice between each pulse, to generate fragments of genomic DNA ranging from 200 to 800 bp in length. For the ChIP assays, equal amounts of treated chromatin were added to microwells containing immobilized antibody for the targeted protein or a negative control normal mouse IgG antibody. In addition, a small portion of treated chromatin, which was equal to 5% of the extracted genomic DNA, was used as the Input DNA to calculate the enrichment of the leptin promoter DNA after immunoprecipitation of the targeted proteins. After incubation for 90 min at 65°C to reverse the cross-links and elute the DNA, Fast-Spin columns were used for DNA purification. The primers for WRN promoter were 5′-CCGCCGCCTGACTTCGGACACC-3′ (forward) and 5′-TCGCACTCCCGCTGCACCCCAC-3′ (reverse).

### 2.8. Cell Culture and Demethylation Treatment

To test the relationship between the methylation and the expression of the WRN, an in vitro study of demethylation was performed. Human lens epithelium cell line (HLEB-3) was obtained from American Type Culture Collection (ATCC; Rockville, MD) and cultured in Eagle's minimum essential medium (Invitrogen-GIBCO, Carlsbad, CA) with 10% fetal bovine serum at 37°C in a humidified 5% CO_2_ atmosphere. After reaching 80–90% confluency, the cells were demethylated by incubation in medium containing 3 mM of 5-aza-2-deoxycytidine (5-aza-dC) (Sigma, CA, USA) for 72 h. Whole cell protein extracts of HLEB-3 were isolated for Western blotting.

### 2.9. Statistical Analysis

One-way ANOVA analysis was used to determine the difference in averages between the four groups. *P* value <0.05 was considered statistically significant. Statistical analyses were performed with SPSS software (SPSS 17.0; SPSS, Inc., Chicago, IL).

## 3. Results

### 3.1. Expression of WRN in Anterior Lens Capsules and Lens Cortex

**Figure 1:**
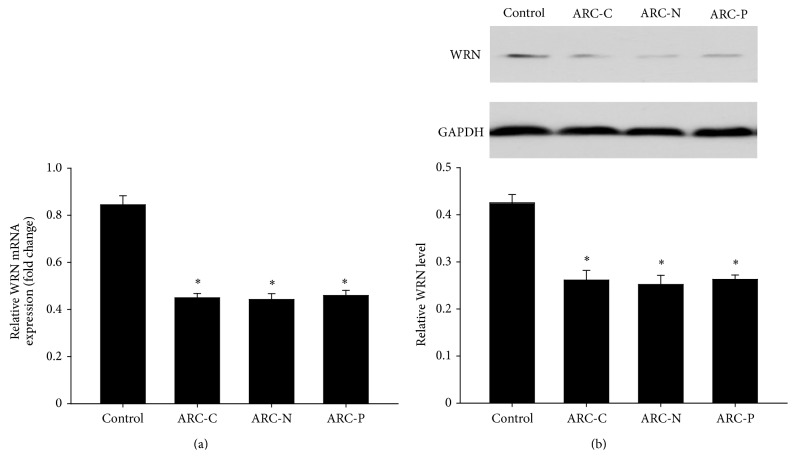
Relative expression of mRNA and protein levels of WRN in anterior lens capsules of control and ARC. (a) RT-PCR analysis of the expression of WRN in control (*n* = 20) and ARC-C (*n* = 20), ARC-N (*n* = 20), and ARC-P (*n* = 20). Values represent mean ± SD. ^∗^ *P* < 0.01. (b) WRN protein levels in control (*n* = 9) and ARC-C (*n* = 9), ARC-N (*n* = 9), and ARC-P (*n* = 9) anterior lens capsules were detected using Western blotting. Relative WRN protein level to GAPDH is presented as mean ± SD. ^∗^ *P* < 0.01.

RT-PCR analysis was performed to investigate WRN mRNA content in anterior lens capsules and context of the ARC and the controls. Lower WRN mRNA (Figure 1(a)) expression in anterior lens capsules was detected in all three subtypes of ARC cases compared with the controls (*P* < 0.01).

To confirm the change of WRN in protein level between ARC and controls, Western blot analysis was performed. As shown in Figure 1(b), the expression pattern of WRN protein in anterior lens capsules was lower in all three subtypes of ARC cases compared with the controls (*P* < 0.01). However, WRN was undetected in lens cortex in both the ARC cases and the controls (data not shown).

### 3.2. Methylation Status of WRN

**Figure 2:**
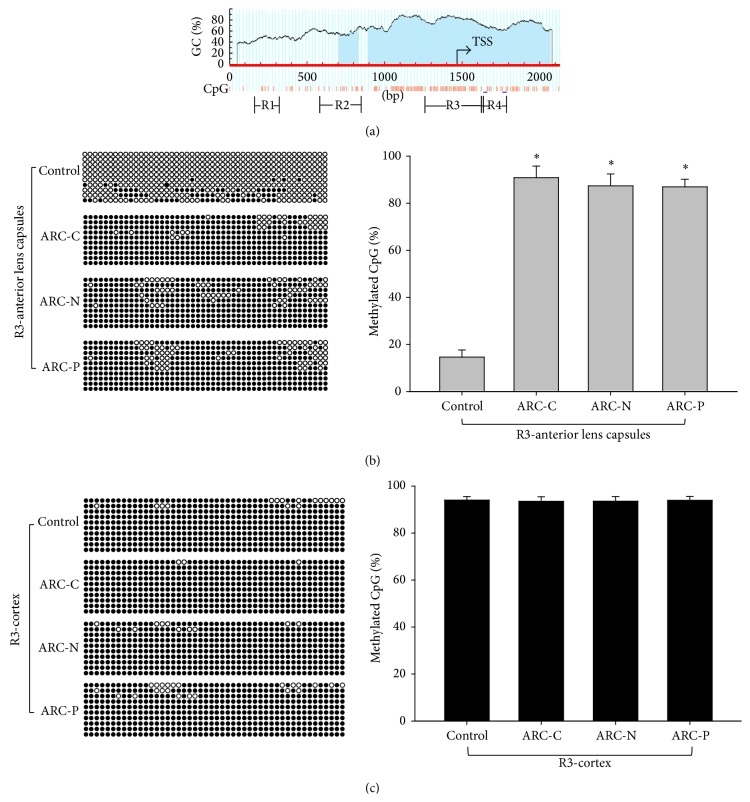
Methylation status at region 3 in WRN promoter in anterior lens capsules and cortex of control and ARC. (a) The positions of CpG islands within WRN promoter. In the following panels, each row of circles represents a single clone. Open and close circles represent unmethylated and methylated CpG sites, respectively. (b) Methylation status of region 3 in WRN promoter in anterior lens capsules of control (*n* = 20) and ARC-C (*n* = 20), ARC-N (*n* = 20), and ARC-P (*n* = 20). The ARC group displayed hypermethylation compared to the control group. ^∗^ *P* < 0.01. (c) Methylation status of region 3 in WRN promoter in lens cortex of control and ARC lens. There were no significant statistical differences between the ARC group and the control group. *P* > 0.05.

To analyze the relationship between methylation status and expression of WRN, we detected the methylation rate of WRN promoter in the DNA extracted from the anterior lens capsules and cortex of ARC and control group using BSP. Bioinformatic analysis indicated four CpG islands in the promoter of WRN (Figure 2(a)) (R1: −1287 to −1133, R2: −875 to −620, R3: −209 to 164, and R4: 173 to 324, relative to the TSS). Figures 2(b) and 2(c) showed a representative result of bisulfite genomic sequencing of the R3 fragment. Each row stands for a single plasmid clone, and each circle represents a CpG site. The unmethylated and methylated CpGs were represented by unfilled and filled cycles, respectively. As shown in Figure 2(b), the methylation rate of all three subtypes of ARC was higher than that of the control group at region 3 of WRN promoter. As shown in Figure 2(c), the methylation rate of lens cortex at region 3 was almost 100% in both ARC group and control group. The methylation rates at regions 1, 2, and 4 in WRN promoter were almost 100% in both ARC group and control group (data not shown).

### 3.3. Histone Modifications around the CpG Islands of the WRN Gene in ARC

**Figure 3:**
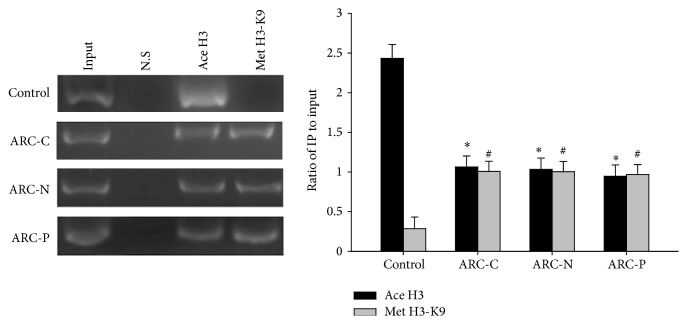
Histone modifications of the WRN promoter in anterior lens capsules of control and ARC. In anterior lens capsules, compared with the control group (*n* = 10), ChIP analysis revealed that acetylated H3 levels were lower in ARC-C (*n* = 10), ARC-N (*n* = 10), and ARC-P (*n* = 10). At the same time, methylated H3-K9 was increased. ^∗^ *P* < 0.01.

In general, hypermethylation of H3-K9 exhibits the silencing of gene expression, whereas acetylation of H3 is associated with activation of gene expression. We performed ChIP to analyze the correlation between the histone modification and the expression profiles of WRN gene. ChIP analysis depicted that acetylated H3 levels were lower in all three subtypes of ARC than the control in anterior lens capsules while methylated H3-K9 was increased in all three subtypes of ARC (Figure 3). The histone modification of cortex was undetectable (data not shown).

### 3.4. Methylation Status and Protein Expression of WRN in HLEB-3 after Treatment with 5-Aza-dC

**Figure 4:**
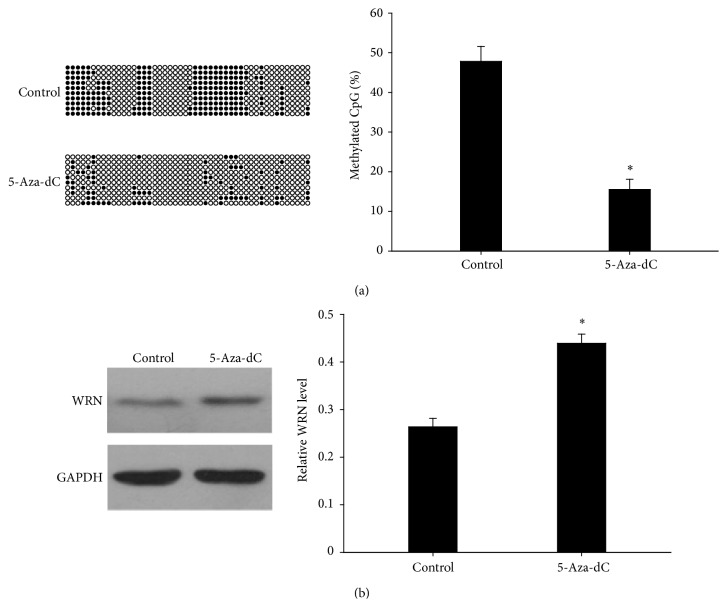
Relative methylation status and expression of protein levels of WRN in HLEB-3 after treatment with 5-aza-dC. In the following panels, each row of circles represents a single clone. Open and close circles represent unmethylated and methylated CpG sites, respectively. (a) After treatment with 5-aza-dC, the methylation rate of WRN in HLEB-3 was decreased. (b) Protein expression of WRN in the untreated control cells and in cells after treatment with 5-aza-dC. Relative WRN protein level to GAPDH is presented as mean ± SD. ^∗^ *P* < 0.01.

To test the relationship between the methylation and the expression of the WRN, an in vitro study of demethylation was performed. 5-Aza-dC is a DNA methyltransferase inhibitor which is used to inhibit DNA methylation. As shown in Figure 4(a), after treatment with 5-aza-dC, the methylation rate of WRN promoter in HLEB-3 was decreased in comparison with the untreated control. In contrast, the protein expression of WRN in HLEB-3 after treatment with 5-aza-dC was increased in comparison with the untreated control (Figure 4(b)).

## 4. Discussion

Although the pathophysiology of ARC is far from being clearly understood, it is well accepted that oxidative stress plays an important role in the disease pathogenesis. When reactive oxygen species (ROS) production exceeds the capacity of its removal by various mechanisms, they may cause oxidative damage to DNA [6–10]. In a normal physiologic condition, most oxidative DNA lesions are rapidly repaired by base excision repair (BER), nucleotide excision repair (NER), and double-strand break repair (DSBR) pathways [13, 14]. WRN is a protein functioning in the DSBR pathway and is also required for cellular DNA replication and mismatch repair [33]. Both mRNA and protein expression of WRN are downregulated in anterior lens capsules in ARC, implying a reduced DNA repair capability in the ARC lens from all included subtypes. The results have provided an additional evidence of DNA repair mechanism in ARC development by using patients' lens tissue.

We demonstrated that WRN undergoes epigenetic alterations in ARC lens tissue from all included subtypes and this alteration is associated with the mRNA and protein expression of the gene. The treatment with a demethylating agent restored the WRN expression in HLEB-3. The results linked the epigenetic changes with the target gene expression and are consistent with the current knowledge on the effect of epigenetic modification on human genome.

It is of interest that the ARC-associated epigenetic changes of WRN gene only occur in anterior lens capsules but not in lens cortex in which both ARC cases and control had an undetectable or very weak expression of WRN and a very high degree of WRN methylation. Lens cortex is made up of lens fibers which are differentiated from LECs. The results suggest that the strategies to intervene epigenetic alteration in ARC should aim at anterior lens capsules.

We analyzed methylation status at four regions of WRN promoter in both ARC and control groups; only region 3 showed significant changes between the cases and the controls. This sequence-specific change is reasonable because this region contains the most abundant CpG islands among the four selected regions and spans the translation starting site.

## 5. Conclusions

Overall, our study found that aberrant epigenetic methylation in WRN DNA and the associated histone linked to low expression of WRN and lens opacity. This is the first report to show a relationship between the epigenetic modification of WRN gene and ARC by directly studying the lens tissue from human subjects. This study provided a deeper insight on the DNA repair mechanism in the pathogenesis of ARC, and the knowledge can be used to identify novel options for the prevention and therapy for ARC.

## Figures and Tables

**Table 1:** Demographic information of study participants.

	Control	ARC
*N*	39	117
Age (mean ± SD)	69.66 ± 4.51	70.38 ± 7.72
Female *N* (%)	21 (54.8)	70 (60.1)
Male *N* (%)	18 (45.2)	47 (39.9)
Cortical *N* (%)	0	39 (33.3)
Nuclear *N* (%)	0	39 (33.3)
PSC *N* (%)	0	39 (33.3)
